# Intercellular GAS6-AXL signaling triggers podocyte cuproptosis in acute kidney injury upstream regulated by CEBPD

**DOI:** 10.1371/journal.pone.0359878

**Published:** 2026-10-09

**Authors:** Jingmei Liang, Yuyun Hu, Bing Liao

**Affiliations:** Department of Nephrology, The First People’s Hospital of Nanning, Nanning, China; IMCB: Institute of Molecular and Cell Biology, SINGAPORE

## Abstract

This study aims to investigate the role of cuproptosis, a novel form of regulated cell death, in the transition from acute kidney injury (AKI) to chronic kidney disease (CKD). Multi-omics approaches, including single-cell RNA sequencing (scRNA-seq), spatial transcriptomics, and in vitro experiments based on cells, were integrated to dissect the spatiotemporal regulation of cuproptosis in AKI. Podocytes exhibited the highest cuproptosis activity. A critical intercellular communication axis was identified, wherein endothelial cell-derived GAS6 signals through the podocyte receptors AXL/TYRO3. The transcription factor CEBPD was upregulated early in AKI and was found to positively regulate the GAS6-related pathway, with its target genes enriched in TGF-β and JAK-STAT signaling, ultimately leading to receptor activation and cuproptosis. Spatially, CEBPD activation preceded the increase in AXL, followed by late-phase upregulation of GAS6. The alteration in AXL protein expression was confirmed in vitro.Our study reveals a CEBPD-mediated transcriptional program that drives podocyte cuproptosis by transactivating the AXL receptor, providing novel mechanistic insights into the AKI-to-CKD transition.

## 1. Introduction

Acute kidney injury (AKI) is a clinical acute and critical condition with impaired renal function leading to multiple complications, which is a common occurrence in major clinical departments and intensive care units (ICUs) [1,2]. More importantly, AKI is extremely likely to progress to chronic kidney injury, and this often results in irreversible damage. Unfortunately, the underlying molecular mechanisms during the transition from AKI to CKD remain poorly understood, hindering the development of effective therapeutic strategies.

In AKI, various forms of regulated cell death (including apoptosis, ferroptosis, etc.) have been proven to be associated with multiple types of kidney cell damage [3,4]. Podocytes, as an important component of the renal filtration barrier, are closely related to the formation of proteinuria and other filtration functions. They are an important cell subset in the progression of AKI. Cuproptosis is a newly discovered type of cell death caused by metabolic disorders of copper ions [5]. Previous studies have shown that patients with Wilson’s disease (copper overload) may initially present with renal injury, highlighting a potential role of disrupted copper homeostasis in kidney damage [6]. During AKI, factors such as ischemia–reperfusion injury, inflammation, and oxidative stress can disturb metal ion homeostasis, leading to abnormal intracellular accumulation of copper ions and thereby creating conditions conducive to the occurrence of cuproptosis. Meanwhile, copper overload can further mediate renal injury—predominantly affecting the renal tubules—by inducing oxidative stress, mitochondrial dysfunction, and metallothionein-associated toxic responses, as well as activating profibrotic pathways [7]. These alterations may manifest as Fanconi syndrome, renal tubular acidosis, and AKI, and in severe cases, may progress to chronic kidney disease [7].

Currently, the research on copper-mediated death mainly focuses on the field of cancer [8,9]. Although some researchers have preliminarily explored the role of copper-mediated death in AKI using bulk tissue transcriptome sequencing data, they were unable to distinguish which specific cell type is driving this process [10]. Furthermore, how cuproptosis is regulated during the process of AKI, and how changes in intercellular communication affect the process of cuproptosis remain unknown.

Hence, to address these gaps, we employed single-cell RNA sequencing (scRNA-seq) to precisely identify the key cell type susceptible to cuproptosis in AKI. Furthermore, we integrated spatial transcriptomics and cellular communication analysis to unravel the intercellular crosstalk that regulates this process. We also sought to identify the upstream transcription factors that orchestrate the cuproptosis-related signaling network.

In this study, we performed a comprehensive multi-omics analysis. Our analysis revealed that podocytes exhibit the highest cuproptosis activity in AKI, revealing that GAS6-AXL/TYRO3 represents a potential key intercellular communication axis. We identified the transcription factor CEBPD as the upstream regulator of this process. Through spatial transcriptomics, we revealed the spatiotemporal dynamics of these key events, and further validated the alterations in AXL protein expression through in vitro cell-based experiments. Our findings unveil a novel regulatory axis that connects transcriptional regulation, intercellular communication, and cuproptosis, providing new insights into the mechanism of AKI progression and potential therapeutic targets.

## 2. Materials and methods

### 2.1. Data download

we obtained the AKI-related single-cell dataset GSE210622 from the Gene Expression Omnibus (GEO) database, comprising 6 control samples and 8 AKI samples [11]. The cuproptosis-related gene set comprises 64 cuproptosis-related genes (CRGs) as reported in the literature by Tao Yan et al [12]. Meanwhile, we used the spatial transcriptome dataset of mouse AKI included in the Kidney Interactive Transcriptomics database (http://humphreyslab.com/SingleCell/) to investigate the spatiotemporal distribution of related genes [13,14].

### 2.2. Quality control of single-cell data using Seurat

We employed Seurat (version 4.4.0) to analyze the 10X data obtained from the single-cell dataset GSE210622 [15]. In addition to the quality control measures mentioned in the original study, we implemented additional filtering criteria, specifically removing cells with low quality based on hemoglobin (< 1%) and ribosome (> 0) levels [16]. Subsequently, we used KNN, Shared nearest neighbor (SNN), Louvain method, referring to Seurat’s best practice standard workflow for data integration (https://satijalab.org/seurat/articles/integration_introduction.html), integrated the data by utilizing functions including NormalizeData, FindVariableFeatures, ScaleData, RunPCA, RunHarmony, and RunUMAP, while referring to the original study for filtering parameters (dims = 1:30, resolution = 0.5).

### 2.3. Single-cell data clustering and annotation

Cell clusters were identified utilizing the recommended cell markers as stated in the original study. Following the identification and removal of injured cells and doublets, we conducted a subsequent data integration using the same methods across all cells. Ultimately, the total cell population of 122,637 cells initially comprised 23 clusters. Through integration and identification processes, these clusters were further consolidated into 12 clusters, resulting in a final count of 111,192 cells.

### 2.4. Intercellular communication

To examine the communication and interaction among all clusters, encompassing signal strength and ligand receptors, we employed the CellChat package (version 1.6.1) [17]. Cellchat objects were generated by combining the processing outcomes and gene expression data from Seurat and subsequently analyzed using the CellChatDB database.

### 2.5. Transcription factor coexpression network

We utilized the SCENIC package (version 1.3.1) to analyze transcription factor-dominated gene regulatory networks, using the hg19-tss-centered-10 kb-10species databases as the reference dataset [18]. Co-expression modules between TFs and candidate target genes were inferred using GENIE3. These co-expression modules were subsequently screened using RcisTarget to identify different regulators. Furthermore, AUCell was employed to score each regulator, while calcRSS was utilized to obtain cell-specific regulators.

### 2.6. Enrichment analysis

We conducted gene enrichment analysis through the online website metaspace(https://metascape.org).

### 2.7. Differential gene expression

We assessed the differential gene expression between the control and AKI groups in various cell subsets using FindMarkers in the scRNA-seq data. We employed a screening condition of “p_val_adj < 0.05” to filter for significant differential genes. Additionally, differential gene analysis in the transcriptome was conducted using the limma package [19]

### 2.8. Gene set enrichment analysis (GSEA)

The clusterProfiler package (version 4.8.3) was utilized to conduct functional enrichment analysis on transcriptome datasets [20]. Visualization of the results was performed using the gseaVis package [21].

### 2.9. Vitro experiments and Western blot

Differentiated Mouse Podocyte Clone-5 (MPC5, MPC5 cells were kindly provided by Cell Bank, Chinese Academy of Sciences.) cells were stimulated with lipopolysaccharide (LPS,1ug/ml) to establish an in vitro podocyte model of AKI. The cells were divided into three groups: control, LPS (15min), and LPS (24h), and treated accordingly. Following the interventions, cells were harvested for Western blot analysis. Total protein was extracted using RIPA lysis buffer, and the protein concentration was determined by the BCA method. The Western blot experiment was conducted using the standard method. Equal amounts of protein were separated by SDS-PAGE and transferred to a PVDF membrane. After blocking, the membrane was incubated with primary antibodies (AXL: Zenbio, Cat#: R23576; β-actin: Zenbio, Cat#: R380624) overnight at 4℃, followed by incubation with HRP-labeled secondary antibody. Signal detection was performed using the ECL chemiluminescence method. The gray value was read using ImageJ and visualized in R.

### 2.10. Statistical analysis

All statistical analyses were conducted using R version 4.3.1. The t-test was employed for normally distributed data, while the Wilcoxon test was utilized for non-normally distributed data. p < 0.05 was considered as a statistically significant difference.

## 3. Result

### 3.1. Work analysis process

S1 File presents a comprehensive overview of the analysis process that integrates single-cell sequencing, spatial transcriptomics, and In vitro experiments based on cells

### 3.2. AKI microlandscapes in single cell data

In our analysis of the GSE210622 dataset, a total of 14 samples were included. After applying the adjusted quality control process, we obtained a final count of 122,637 cells. These cells were subjected to cluster analysis using the Shared nearest neighbor (SNN), resulting in the identification of 23 distinct cell clusters (Fig 1A). The expression levels of major cell type markers mentioned in the original study were used for identification (S1 Table). Based on the cell markers used in the original study, we assigned the following interpretations: clusters 0, 2, and 12 represented the thick ascending limb (TAL), proximal tubule (PT) clusters were divided into 1, 3, 16, and 19, cluster 9 represented the distal convoluted tubule (DCT), clusters 5, 11, and 22 were identified as endothelial cells (EC), clusters 6 and 8 represented the thin limb (TL), clusters 13 and 17 were categorized as interstitial cells (IntC). Additionally, clusters 10 and 21 were assigned as collecting duct intercalated cells type A and B (CD-IC-A/B), cluster 15 referred to leukocytes (LEUK), and cluster 18 corresponded to podocytes (POD). Clusters 14 were identified as connecting tubule (CNT), clusters 4 and 7 were assigned to collecting duct principal cells (CD-PC), while clusters 20 and 23 did not exhibit specific expression genes, thus they were excluded. Subsequently, the included clusters were combined again using the harmony program, which resulted in a total of 111,192 cells (Fig 1B). The composition of cell types in each sample is displayed in a bar chart (Fig 1C). Furthermore, the expression levels of 11 core cuproptosis regulatory genes were significantly altered between the control and AKI groups(Fig 1D).

**Fig 1:**
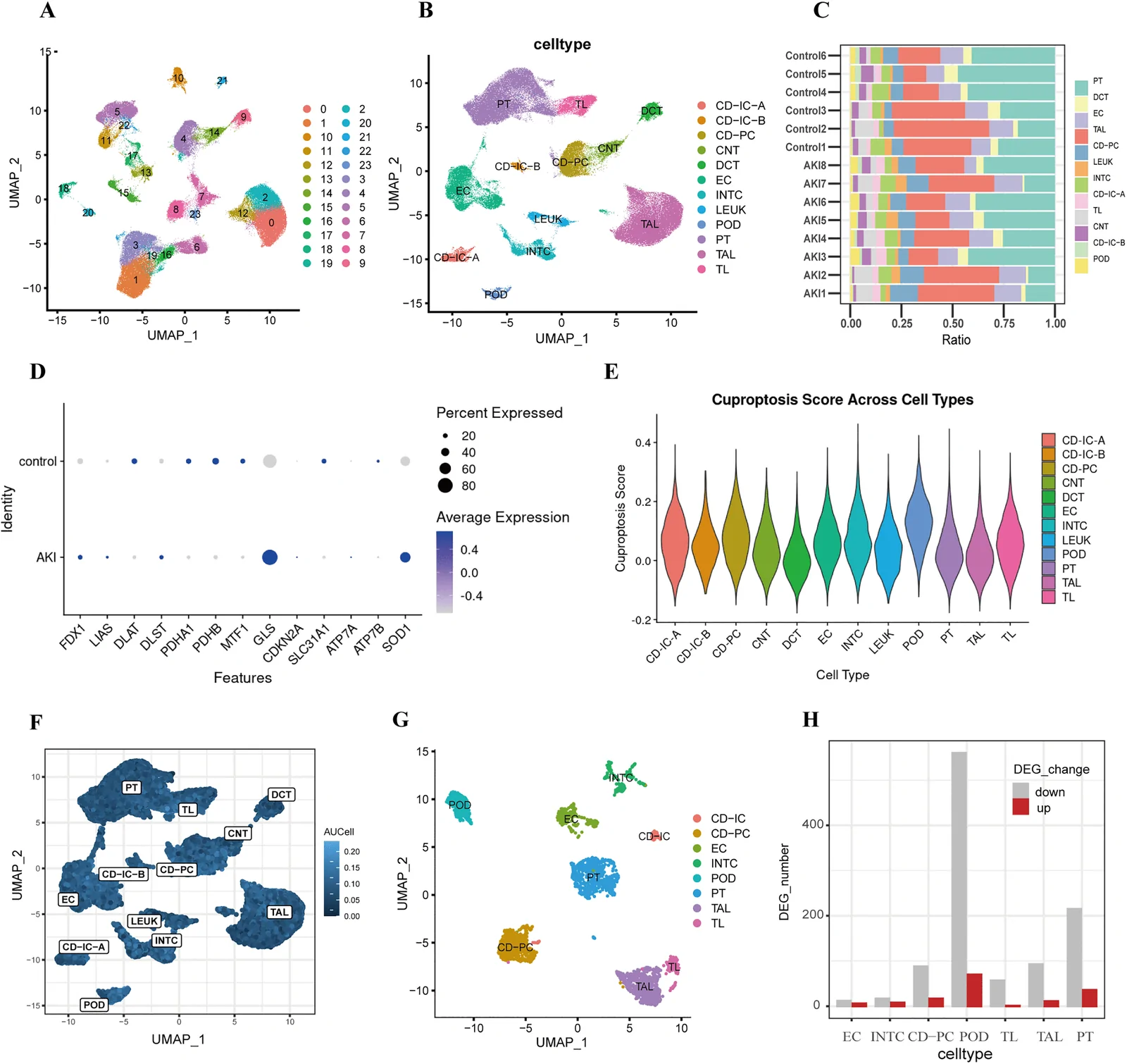
Single-cell landscape and cuproptosis activity in AKI. (A) UMAP visualization cells colored by 23 clusters; (B) UMAP visualization final cells; (C) The proportion of cell types in each sample; (D) Expression Dot Plot of 11 core cuproptosis regulatory genes in podocytes; (E) Cuproptosis scores across major cells (podocytes shown with highest score); (F) UMAP visualization of cuproptosis enrichment scores across all cells; (G) UMAP visualization of 2,747 high cuproptosis-score cells; (H) Bar chart showing transcriptional activity (number of expressed genes) in high-score cells.

### 3.3. Podocytes exhibit the highest cuproptosis activity

We employed AUCell version 1.22.0 to calculate enrichment scores for all cells using a set of 64 CRGs [22]. At the same time, we also scored using the Ferroptosis-related gene set (GPX4、SLC7A11、SLC3A2、GCLC、GCLM、ACSL4、LPCAT3、ALOX12、TFRC、NCOA4、FTH1、FTL、SLC40A1、IREB2、NFE2L2、KEAP1、HMOX1、NQO1、AIFM2、GCH1、TP53、SAT1、ATG5、ATG7、PTGS2、CHAC1), and the relevant results are shown in S1A Fig. Among all cell subgroups, podocytes showed the highest cuproptosis-related enrichment score (Fig 1E). Moreover, the relative enrichment of the cuproptosis-related transcriptional signature in podocytes was significantly greater than that of the ferroptosis-related signature (S1B Fig). Cells with enrichment scores exceeding 0.15 were defined as “high-score cells” (Fig 1F). To visualize the high-score cells, we applied the UMAP method and conducted cluster analysis. Subsequently, we identified and grouped the high-score cells based on the cell markers described in the original study, while also removing any cells exhibiting bimodal expression. Notably, the CD-IC-A and CD-IC-B populations were merged into the CD-IC group, resulting in a total of 2747 high-score cells (Fig 1G). Interestingly, we found that the transcriptional activity of podocytes was the most active in the high-score cell population (Fig 1H). These results pinpointed podocytes as the most vulnerable cell type to cuproptosis in AKI, providing a focus for subsequent mechanistic investigations

### 3.4. The GAS pathway mediates endothelial-podocyte crosstalk

CellChat was employed to infer intercellular communication among distinct cell subpopulations within the high-scoring cell population, with a particular focus on predicting potential ligand–receptor interactions. Fig 2A provides a comparison of the number and strength of cell communication connections. In comparison to the con, AKI demonstrated a lower number of connections, while the communication strength between the two groups remained relatively similar. Fig 2B indicates that in the relative information flow of intercellular communication, the VISFATIN, GAS, and TGF-β pathways were specifically activated in the AKI group. Further examination of the activation status of these three pathways reveals that the GAS pathway is the most significantly activated in the podocytes(Fig 2C). By expanding the signaling network of the GAS pathway, we found that the signals were mainly emitted by endothelial cells, while podocytes and mesenchymal cells were the main recipients. Further analysis of the ligand-receptor pairs revealed that GAS6-TYRO3 and GAS6-AXL were the main contributors(Fig 2D). Finally, we found that in endothelial cells, the expression level of GAS6 was lower than that in the control group, while in podocytes, the expression levels of AXL and TYRO3 were slightly higher than those in the control group, whereas MERTK showed minimal expression in both groups. This suggests that the GAS6-AXL/TYRO3 ligand-receptor pair serves as a critical communication channel from endothelial cells to podocytes, potentially regulating cuproptosis.

**Fig 2:**
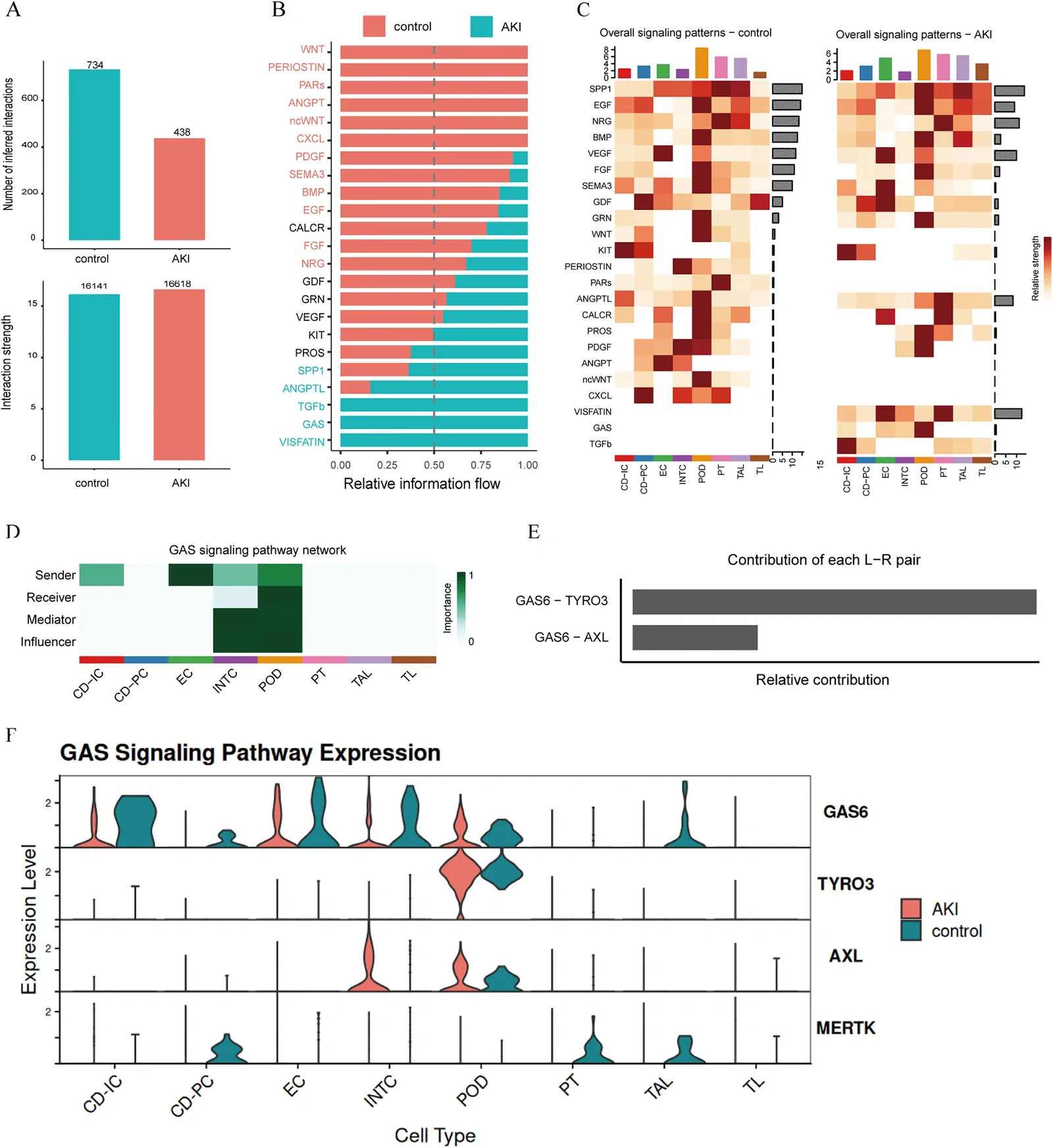
CellChat analysis identifies the activated GAS6-AXL/TYRO3 axis in cuproptosis-high cells during AKI. (A) Comparison of the number and weight of intercellular interactions between; (B) CellChat predicts differences in relative information flow between groups within the high-scoring cell populations.; (C) CellChat predicts differences in signaling pathway strength among distinct cell subpopulations within the high-scoring cells.; (D) The communication network of the GAS signaling pathway; (E) The dominant ligand-receptor pairs contributing to the GAS pathway; (F) The violin plot shows the expression of the GAS signaling pathway and its core ligand receptors.

### 3.5. CEBPD acts as an regulator of signaling

The SCENIC tool was utilized to analyze high-score cells and infer TFs networks. The results showed that compared with the control group, the activities of the five transcription factors (CEBPD, etc.) were significantly increased in the AKI group(Fig 3A). The expression levels of CEBPD and its regulatory factors were all found to increase in the AKI group(Fig 3B). To identify podocyte-specific regulators, we performed an additional SCENIC analysis on podocyte subpopulations.Although the CEBPD target genes showed no direct overlap with canonical cuproptosis genes, GSEA revealed that CEBPD target genes were significantly enriched in gene sets related to GAS6-associated pathways (NES = 2.18, p < 0.001) (Fig 3C).KEGG enrichment analysis further revealed that these core genes are closely related to the TGF-β and JAK-STAT pathways(Fig 3D). This indicates that while CEBPD does not directly regulate the execution of cuproptosis, it orchestrates a transcriptional program that potently activates pathways associated with cell communication, thereby bridging cuproptosis to intercellular signaling.

**Fig 3:**
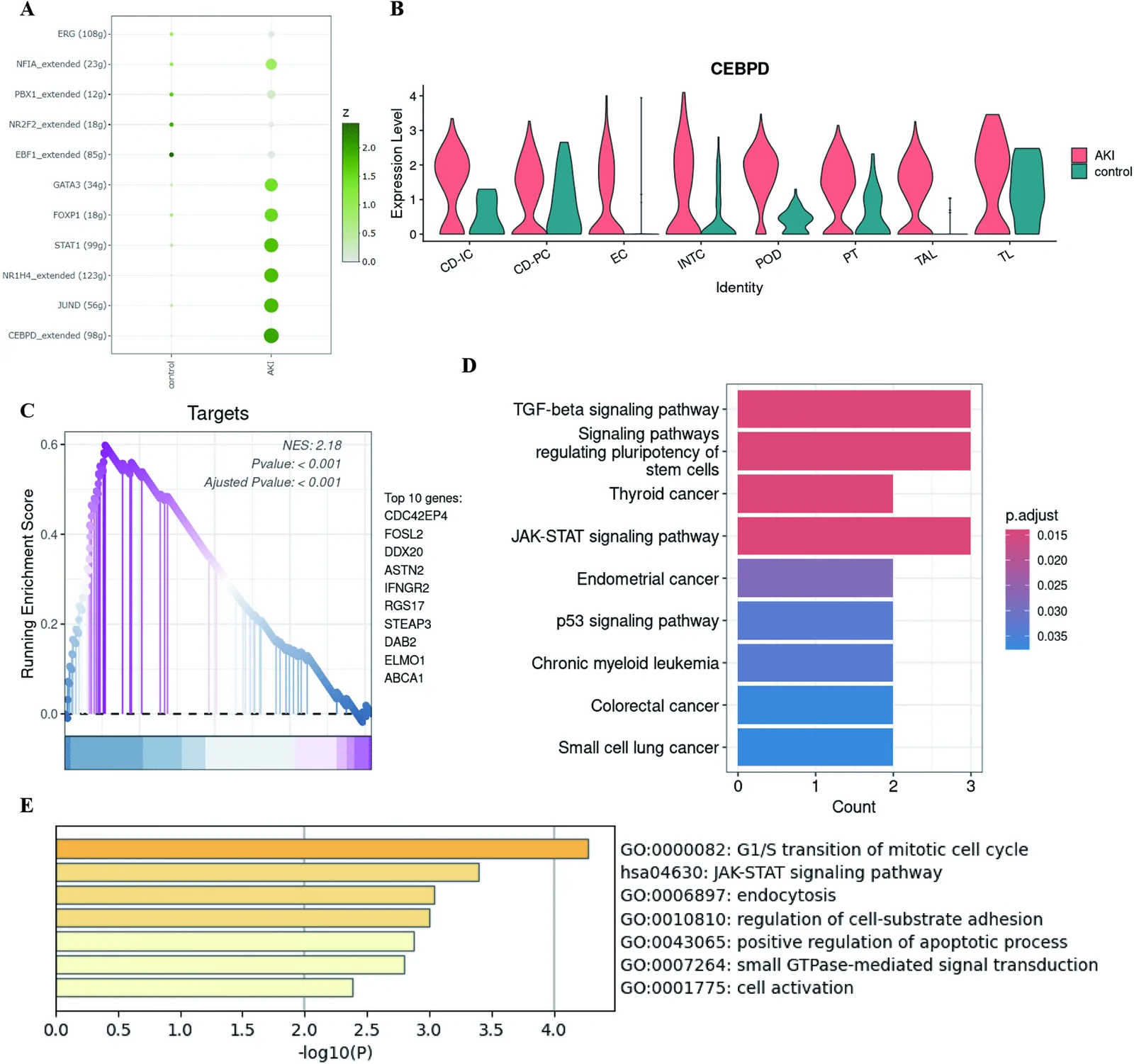
The transcription factor CEBPD is activated and regulates the pro-damage network. (A) Transcription factor activity analysis reveals top five upregulated TFs in AKI; (B) Violin plots shows the expression of CEBPD in all cells; (C) GSEA shows significant enrichment of CEBPD target genes in GAS6-associated pathway gene sets. (D) KEGG pathway enrichment analysis of CEBPD core target genes; (E) Functional enrichment analysis (GO Biological Processes) of core CEBPD core target genes.

We further extracted the core genes from the CEBPD target gene set and conducted an enrichment analysis on the Metaspace website. The results showed that the core genes of the CEBPD target genes are closely related to the regulation of cell cycle and cell apoptosis (Fig 3E). This places CEBPD as an upstream master regulator that activates a pro-fibrotic signaling network, which in turn may lead to the upregulation of the AXL receptor.

### 3.6. Spatiotemporal validation of a regulatory cascade

We utilized the spatial transcriptomic data on mouse ischemia-reperfusion provided by the Kidney Interactive Transcriptomics website to validated the temporal cascade of key events: CEBPD activation (4h) preceded the upregulation of its potential downstream target, AXL (12h), which was followed by a late-phase induction of GAS6 (6w).And TYRO3 expression changed little throughout the process, with only significant changes observed in URO cells (Fig 4A-4D).

**Fig 4:**
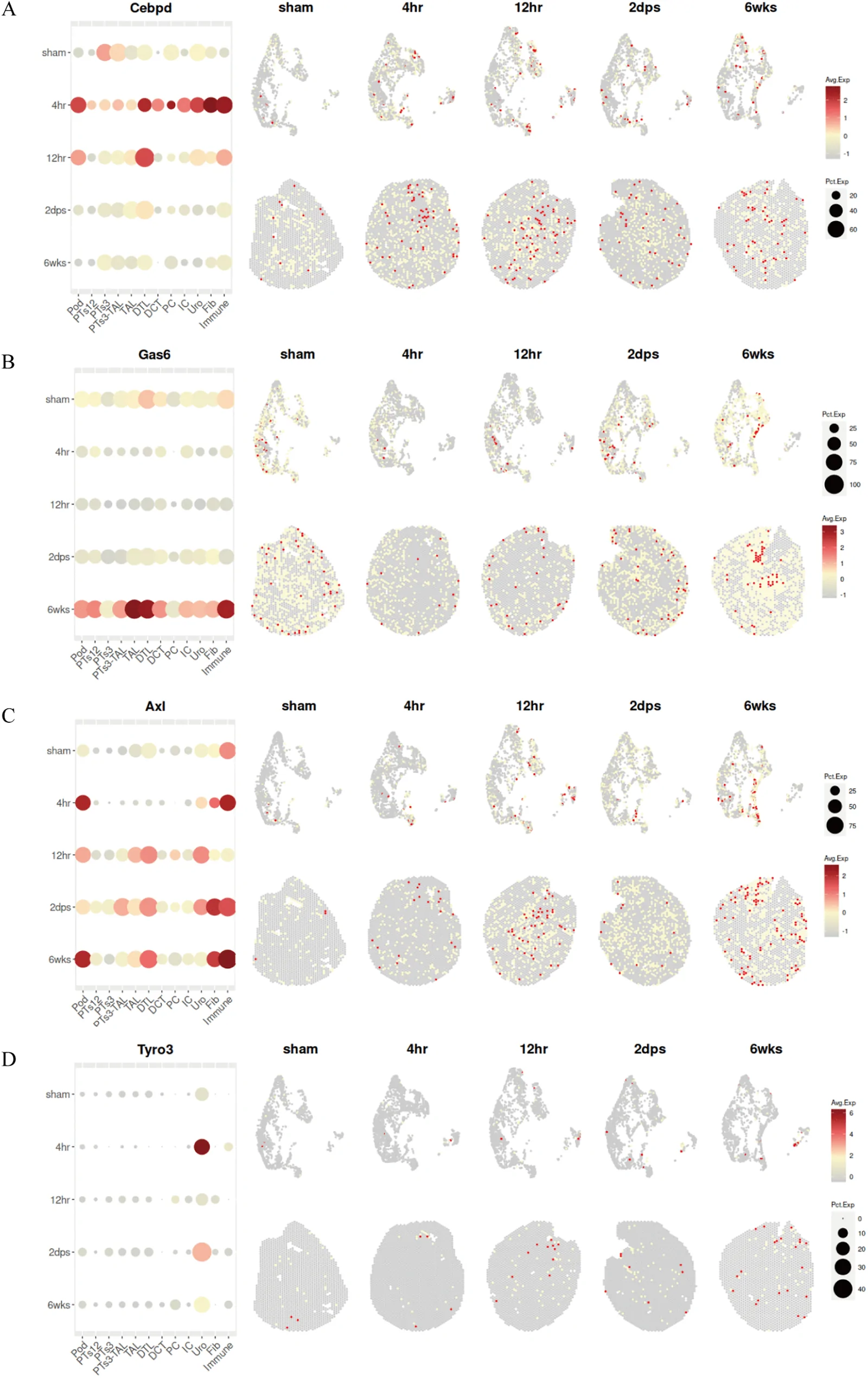
Spatiotemporal transcriptomics validates the sequential activation of the CEBPD-AXL-GAS6 axis. (A-D)Spatial expression pattern of CEBPD(A)、GAS6(B)、AXL(C)、TYRO3(D).

### 3.7. Vitro experiments verified the expression of AXL

The results of Western Blot are shown in Fig 5 and S2 Fig. Quantitative analysis showed that AXL expression was significantly increased in the LPS(24h) group compared with the control group (P = 0.03 < 0.05).

**Fig 5:**
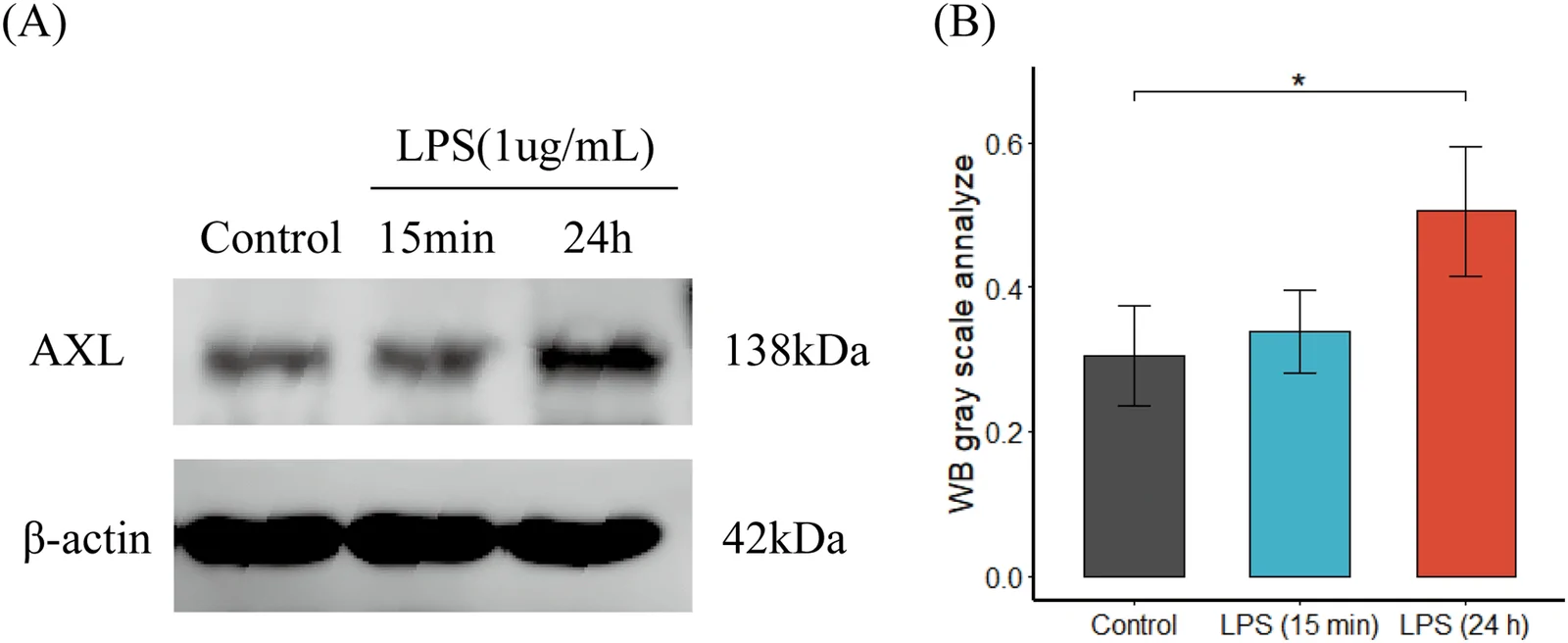
The in vitro experimental verification results of AXL. (A) The expression of AXL with LPS in MPC5 (B) Analysis of the gray values (The data are presented as mean ± standard deviation (mean ± SD) (n = 3),*P < 0.05).

## 4. Discussion

In this study, we employed a multi-omics approach to reveal a novel molecular pathway that links cuproptosis to renal injury. We found that podocytes are the cell type most susceptible to cuproptosis in acute kidney injury. Subsequently, we discovered that podocytes affected by cuproptosis undergo a series of changes mediated by the increased activity of transcription factor CEBPD. These changes promote the upregulation of AXL receptor expression, which combines with the GAS6 signal produced by endothelial cells. This mechanism was verified in the podocyte model of acute kidney injury, suggesting a potential mechanism underlying the progression from acute kidney injury to chronic kidney disease.

Among the key players in this axis, the transcription factor CEBPD represents a particularly intriguing finding. Previous studies have observed that the expression level of CEBPD is elevated in the pathology of acute kidney injury, and it is also related to the inflammatory response [23,24]. However, the role of CEBPD in cuproptosis has not yet been reported. Our research has revealed that cuproptosis serves as a novel trigger for activating CEBPD in the podocytes of acute kidney injury. Although the target genes regulated by CEBPD do not directly participate in the regulation of the cuproptosis process, through GSEA enrichment analysis, we found that the target genes regulated by CEBPD were significantly enriched in the GAS6-related pathway. Among them, the core target genes are closely related to the TGF-β and JAK-STAT pathways, which have been recognized as being closely associated with AKI and CKD [25–27]. These findings strongly suggest that CEBPD may convert the damage caused by cuproptosis into signals that promote fibrosis and inflammation.It has been implicated in the pathogenesis of fibrosis in diverse organs, including the lungs and lens [28]. This aligns with and strengthens our findings, suggesting that CEBPD may act as a conserved regulator of fibrotic processes across tissue types. Therefore, CEBPD may act as a central orchestrator of fibrotic signaling, integrating upstream damage cues to drive the expression of a pro-fibrotic gene repertoire.This suggests a complex, context-dependent role for CEBPD and its downstream signals, prompting us to conduct further research on its more profound regulatory mechanisms.

One of the most critical downstream effects of this CEBPD-driven pro-fibrotic program appears to be the dysregulation of the GAS6-AXL signaling axis. The GAS6-AXL signaling axis, a key member of the TAM receptor tyrosine kinase family, plays a central role in inflammatory states, apoptosis, and glomerular mesangial proliferation [29]. We found that the GAS6-AXL axis is activated in the early phase of AKI, a finding that is strongly consistent with the work of Chen.D Q et al [30], who demonstrated that the initial upregulation of GAS6-AXL signaling constitutes a protective response to mitigate oxidative stress and inflammation following renal injury.Integrating our data with the findings of Chen.D Q et al, we propose that the GAS6-AXL pathway may plays a biphasic or context-dependent role in kidney injury. The cytoprotective function of AXL signaling during the acute phase is powerfully underscored by Hyde G.D et al [31]. They showed that AXL knockout mice subjected to renal ablation exhibited significantly exacerbated tubular injury and apoptosis, despite upregulation of the ligand GAS6. This indicates that the observed upregulation of the GAS6-AXL axis in injury models represents a compensatory survival response—the system attempts to protect itself, and removal of this safeguard leads to worsened outcomes.

AXL expression is dysregulated in renal diseases, which were robustly corroborated by immunohistochemical evidence from human kidney biopsies. Fiebeler,A et al comprehensively analyzed 26 human kidney specimens from patients with various immune-mediated glomerulonephritides, including IgAN and LN [32]. They reported that AXL and GAS6 were nearly undetectable in normal kidneys but were markedly upregulated in diseased tissues, with AXL abundantly expressed in glomeruli and tubules, and GAS6 upregulated in vasculature, tubules, and glomeruli. Our in vitro experiments demonstrated that AXL is upregulated under AKI conditions, consistent with previous findings, suggesting that dysregulation of the GAS6–AXL axis may represent a hallmark of human inflammatory kidney diseases.

Based on these findings, we hypothesize that during the acute stage of kidney injury, the upregulation of AXL may contribute to cellular protection, potentially mitigating acute tissue damage and inflammatory responses. However, the persistent activation of this pathway into the chronic phase, may maladaptively promote fibrotic and inflammatory processes, thereby driving the progression to CKD.

At present, only a small number of AKI patients are able to recover fully. Even if patients recover after experiencing AKI, the risk of developing CKD remains significantly high [33]. Our research has revealed that the damage to the CEBPD-AXL axis series mediated by cuproptotic might be a potential driving factor for this transformation. Previous studies have reported concurrent increases in urinary albumin excretion and GAS6–AXL expression in diabetic rat models [34], strongly suggesting that this mechanism may represent another potential pathway driving the transition from acute kidney injury to chronic disease.

In addition to elucidating a new mechanism of AKI injury, our findings also provide potential scientific basis for improving the clinical treatment plans for AKI patients. AXL can detach and enter the circulatory system. Taking advantage of this characteristic, it can be used as a diagnostic marker or a risk stratification marker. Currently, AXL is mainly regarded as a target for tumor treatment. Corresponding targeted inhibitors have been developed and have demonstrated certain safety in clinical trials.

The potential of AXL-targeted therapy as a novel treatment for AKI is highly promising. At present, inhibitors targeting AXL have been developed and have shown certain safety in clinical trials. Although AXL has both positive and negative aspects in our study, the treatment prospects are still promising. More importantly, this idea has received significant preclinical evidence: The research conducted by Kurata,A et al. revealed that the use of AXL blockers could alleviate kidney damage in mice caused by hypertension and reduce albuminuria and even mitigate epithelial-mesenchymal transition [35,36]. The biphasic nature of the GAS6-AXL signaling is of great significance for the development of temporally targeted treatment strategies. The chronic stage of injury may be the optimal time to use AXL inhibitors to prevent AKI from completely progressing to CKD. This concept was supported by the subsequent observations of the authors, namely: downregulation of this pathway can weaken the process of fibrosis. Therefore, targeting the CEBPD-GAS6-AXL axis, especially AXL, represents not only a scientifically grounded but also a highly promising therapeutic strategy for preventing and treating the transformation of AKI into chronic kidney disease. In the future, through renal biopsy or serum biomarker detection, it will be possible to identify the AKI subgroups characterized by high expression of AXL or activation of CEBPD. These individuals will become the ideal target population for AXL-targeted therapy, thereby achieving the maximum clinical benefits.

Despite the compelling evidence, our study has several limitations. First and foremost, the predominantly bioinformatic nature of our study establishes strong associations but cannot definitively prove causality. Future studies employing podocyte-specific knockout models are essential to confirm the causative role of this axis. Second, the precise molecular mechanism by which CEBPD regulates AXL (e.g., direct transcriptional activation) remains to be fully elucidated through experiments such as ChIP-seq. Third, integrating data from diverse platforms may introduce technical artifacts, although our focus on consistent trends across datasets strengthens the conclusions. These limitations, however, do not detract from the main findings but rather provide a clear roadmap for future mechanistic investigation.

In conclusion, our integrative analysis unveils a previously unrecognized CEBPD-GAS6-AXL axis that is initiated by cuproptosis in podocytes and may critically contribute to the maladaptive response following AKI, ultimately promoting fibrosis and CKD progression. Targeting this axis, particularly AXL, represents a promising novel therapeutic strategy for preventing AKI-to-CKD transition.

## Supporting information

S1 Table The cell markers used in this study. (XLSX)

S1 Fig Comparison of two cell death scores (A) The scoring situation (after standardization) of the two types of cell death in all cell types (B) Comparison of the scores for the two types of cell death in the podocytes(after standardization). (TIF)

S2 Fig Original gel image. (TIF)

S1 File Graphical abstract. (TIF)
